# The AGC Kinase Inhibitor H89 Attenuates Airway Inflammation in Mouse Models of Asthma

**DOI:** 10.1371/journal.pone.0049512

**Published:** 2012-11-26

**Authors:** Laurent L. Reber, François Daubeuf, Simona Nemska, Nelly Frossard

**Affiliations:** Laboratoire d’Innovation Thérapeutique, UMR 7200 CNRS-Université de Strasbourg, Faculté de Pharmacie, Illkirch, France; French National Centre for Scientific Research, France

## Abstract

**Background:**

H89 is a potent inhibitor of Protein Kinase A (PKA) and Mitogen- and Stress-Activated protein Kinase 1 (MSK1) with some inhibitory activity on other members of the AGC kinase family. H89 has been extensively used *in vitro* but its anti-inflammatory potential *in vivo* has not been reported to date. To assess the anti-inflammatory properties of H89 in mouse models of asthma.

**Methodology/Principal Findings:**

Mice were sensitized intraperitoneally (i.p.) to ovalbumin (OVA) with or without alum, and challenged intranasally with OVA. H89 (10 mg/kg) or vehicle was given i.p. two hours before each OVA challenge. Airway hyperresponsiveness (AHR) was assessed by whole-body barometric plethysmography. Inflammation was assessed by the total and differential cell counts and IL-4 and IL-5 levels in bronchoalveolar lavage (BAL) fluid. Lung inflammation, mucus production and mast cell numbers were analyzed after histochemistry. We show that treatment with H89 reduces AHR, lung inflammation, mast cell numbers and mucus production. H89 also inhibits IL-4 and IL-5 production and infiltration of eosinophils, neutrophils and lymphocytes in BAL fluid.

**Conclusions/Significance:**

Taken together, our findings implicate that blockade of AGC kinases may have therapeutic potential for the treatment of allergic airway inflammation.

## Introduction

Asthma is a very common chronic inflammatory disease affecting over 300 million people worldwide [1] and its prevalence is rising [2]. Although most patients respond very well to current therapies, including corticosteroids and β2-agonists, about half of them still report episodes of uncontrolled asthma [3]. Moreover, a small portion (5–10%) of asthmatic patients fails to respond to corticosteroids [1], [4], [5] highlighting a need for new therapies. It has been proposed that enhanced kinase activity could be responsible, at least in part, for this corticosteroid resistance [5], [6], [7].

Because asthma is a very complex inflammatory disease, involving a broad spectrum of cytokines, chemokines and other inflammatory mediators [8], [9], it is unlikely that targeted inhibition of a single molecule or receptor might result in an effective treatment [1]. Indeed, the efficiency of corticosteroids is based on repression of many transcription factors [10]. Kinases play a major role in regulating the expression of inflammatory genes in asthma [7] and kinase inhibitors are now in preclinical development for the treatment of inflammatory diseases, including asthma [1], [7].

*N*-[2-(*p*-Bromocinnamylamino)ethyl]-5-isoquinolinesulfonamide) (H89) is a potent inhibitor of the cAMP-dependent Protein Kinase A (PKA) [11] and Mitogen- and Stress-activated Kinase 1 (MSK1) [12], [13], and shows some selectivity for other members of the AGC kinase family, including p70 ribosomal protein S6 kinase 1 (S6K1) and Rho-associated kinase (ROCK)-II [14], [15]. Both PKA and MSK1 can activate transcription factors implicated in inflammatory gene expression, including NF-κB [12], [13], [16], [17] and CREB [18], [19], [20], [21].

A previous report indicates that H89 can inhibit IL-5 promoter activity and IL-5 expression in Th2 cells *in vitro* [22]. Considering the central role of IL-5 in eosinophil biology and in the pathophysiology of asthma [23], we investigated the effect of H89 in mouse models of asthma [24], [25].

## Materials and Methods

### Mice

Male BALB/c and C57BL/6 mice were purchased from Charles River Laboratories. Animals were maintained under controlled environmental conditions with a 12 h/12 h light/dark cycle according to the EU guide for use of laboratory animals. Food (UAR-Alimentation) and tap water were available *ad libitum*. Animal experimentation was conducted with the approval of the local ethics committee that regulates animal research at the University of Strasbourg (‘*Comité Régional d’Ethique en Matière d’Expérimentation Animale de Strasbourg’* (CREMEAS)).

### Allergen Sensitization and Challenge

#### Acute asthma model

Nine week-old BALB/c mice were sensitized (i.p.) on days 0 and 7 with 50 µg chicken egg albumin (OVA, Grade V) adsorbed on 2 mg aluminium hydroxide (alum) in saline (23918-6, Sigma-Aldrich). Control animals received i.p. injections of alum in saline only. Mice were challenged on days 18, 19, 20 and 21 by intranasal (i.n.) instillations of 10 µg OVA in saline or with saline alone for controls (12.5 µl/nostril). These challenges were performed under anesthesia (i.p.) with 50 mg/kg ketamine (Imalgene®, Merial) and 3.33 mg/kg xylazine (Rompun®, Bayer).

#### Moderate asthma model

9 week-old C57BL/6 mice were sensitized i.p. on days 0 and 7 with 50 µg OVA or with saline for control animals. Mice were challenged (i.n.) on days 47, 50 and 53 with 10 µg OVA in saline or saline alone for control animals.

### Treatment with H89

H89 (*N*-[2-(*p*-Bromocinnamylamino)ethyl]-5-isoquinolinesulfonamide], di-HCl Salt) (10 mg/kg) (LC Laboratories, PKC Pharmaceuticals Inc., Woburn, MA, USA) suspended in 5% DMSO in saline was administered i.p. two hours before each OVA challenge (or two hours before the last OVA challenge only for Figure S3). Control animals received equivalent volumes (200 µl) of 5% DMSO in saline.

### Measurement of Airway Responsiveness

Airway responsiveness to aerosolized methacholine (MCh) (Sigma Chemicals) at increasing concentrations was measured 24 hours after the last OVA challenge (on day 22 in the acute model and on day 54 in the moderate model) by whole body barometric plethysmography (Emka Technologies, Paris, France) [26]. As previously reported [27], [28], mice were stabilized in the plethysmograph chamber for 30 min until stable baseline, and then exposed to aerosolized saline (30 sec) as a control. Mice were then challenged every 20 min with aerosolized MCh (0.05, 0.1, 0.2 and 0.3M) for 30 sec each, and the enhanced pause (PenH) was recorded during 5 min and used as an index of airway obstruction.

### Total and Differential Cell Counts in Bronchoalveolar Lavage (BAL) Fluid

BAL and differential cell counts were performed as previously reported [27], [28]. Briefly, mice were anaesthetized i.p. (Ketamine 50 mg/kg – Xylasine 3 mg/kg). After semi-excision of the trachea, a plastic canula was inserted, and airspace washed with 0.5 ml of 0.9% NaCl injected with a 1 ml syringe. This operation was performed 10 times. The initial concentrated supernatant of the 2 first lavages (volume = 2×0.5 ml administered, ∼0.5 ml back) was collected for cytokine measurements. The rest of the bronchoalveolar lavage was centrifuged (600 *g* for 10 min, 4°C), and cell pellets pooled. After lysis of erythrocytes with distilled water followed by osmotic re-equilibration, the cell pellet was suspended in 500 µl of 0.9% NaCl and used for total cell counts on a hemocytometer chamber. For differential cell counts, cells were cytocentrifuged at 700 rpm for 10 min (Shandon cytospin), and labelled with Diff-Quick® staining. Differential cell counts on at least 400 cells were obtained using standard morphological criteria.

### Histological Analysis

Lung tissues were fixed (4% paraformaldehyde) and paraffin-embedded. 6-µm sections were cut, mounted on Superfrost glass slides (Fischer Scientific), and stained with H&E or periodic acid-Schiff (PAS) or toluidine blue (all from Sigma-Aldrich). To determine the severity of the inflammatory cell infiltration, peribronchial cell counts were performed based on a 5-point scoring system described by Myou *et al*. [29]. The extent of mucus production was quantified using a 5-point grading system described by Tanaka *et al*. [30].

### ELISAs

IL-4, -5 and -10 were quantified in BAL fluids collected 24 h after the last OVA challenge using ELISA kits (BD Pharmingen) according to the manufacturer’s instructions. OVA-specific IgE, IgG1, IgG2a (for Balb/c mice) and IgG2c (for C57BL/6 mice) serum levels were determined by ELISA as previously described [31].

### Statistical Analysis

Data are presented as means ± SEM. Differences in airway responses between different groups were statistically analyzed using a two-way ANOVA followed by a Bonferroni post-test. For all other experiments, statistical differences were analyzed using Student’s *t* test. Data were considered significantly different when *P*<0.05.

## Results

### Effect of H89 on OVA-induced Airway Hyperresponsiveness

**Figure 1:**
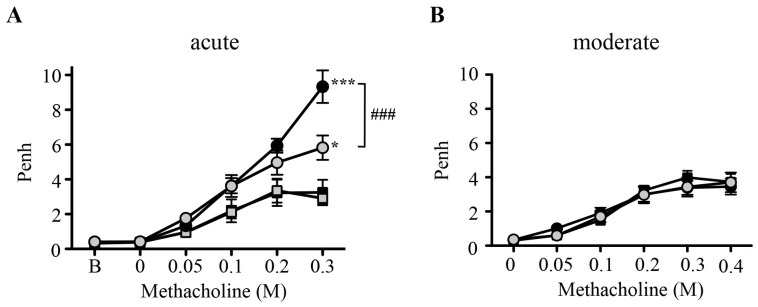
Effect of H89 on the development of airway hyperreactivity in the acute asthma model. A–B. Penh responses to aerosolized methacholine in control mice (square) and OVA-sensitized/challenged mice (circle) treated with vehicle (black) or H89 (10 mg/kg) (grey) in the acute (**A**) and moderate (**B**) asthma models. (B = baseline). Data represent mean values ± SEM (bars) from *n* = 6−9 mice for control groups and *n* = 12 mice for OVA sensitized/challenged groups (OVA). *P<0.05 and ***P<0.001 *vs* corresponding controls; ^###^ *P*<0.001 *vs* group indicated; NS: not significant.

OVA-sensitization and subsequent challenge is known to lead to the development of airway hyperresponsiveness (AHR) in the acute asthma model. We therefore assessed the effect of the AGC kinase inhibitor H89 (10 mg/kg, administered i.p. 2 h before each challenge) on airway responses to aerosolized methacholine (MCh) by a non-invasive method measuring the enhanced pause [Penh] at 24 h after the last challenge. As expected, OVA sensitized/challenged BALB/c mice exhibited increased Penh responses to MCh as compared to saline-treated mice. Treatment with H89 significantly inhibited AHR in OVA sensitized/challenged mice, whereas it had no effect on airway responses in control mice (**Fig. 1A**). By contrast, OVA sensitized/challenged C57BL/6 mice did not develop AHR in the moderate asthma model (**Fig. 1B**).

### Effect of H89 on OVA-induced Inflammatory Cell Influx in BAL Fluid

**Figure 2:**
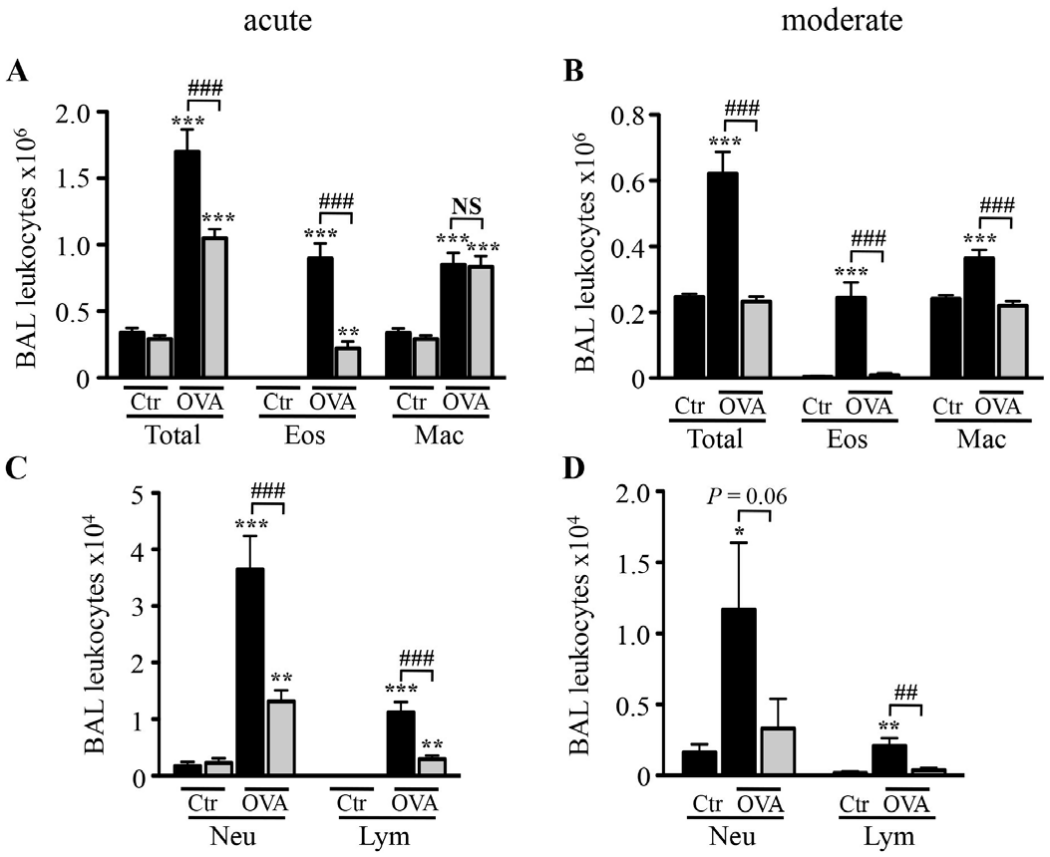
Effect of H89 on numbers of leukocytes in BAL fluid. A–B. Effect of H89 (10 mg/kg) (grey blocks) or vehicle (black blocks) on total leukocyte (Total), eosinophil (Eos) and macrophage (Mac) numbers in BAL fluid 24 hours after the last challenge in control (Ctr) and OVA-sensitized/challenged mice (OVA) in the acute (**A**) and moderate (**B**) asthma models. **C–D.** Effect of H89 (10 mg/kg) (grey blocks) or vehicle (black blocks) on neutrophils (Neu) and lymphocytes (Lym) numbers in BAL fluid 24 hours after the last challenge in control (Ctr) and OVA-sensitized/challenged mice (OVA) in the acute (**C**) and moderate (**D**) asthma models. Data represent mean values (blocks) ± SEM (bars) from *n* = 6−9 mice per group (saline) and *n* = 9−12 mice per group (OVA). *P<0.05, **P<0.01 and ***P<0.001 *vs* corresponding controls; ^##^ *P*<0.01 and ^###^ *P*<0.001 *vs* group indicated; NS: not significant.

OVA sensitized/challenged mice displayed a significant increase in total cell infiltrate in BAL fluid in both models as compared to control mice (5.1-fold and 2.5-fold for the acute and the moderate model, respectively) (**Fig. 2A & 2B**). In the acute model, this cell infiltrate consisted of 50.1% eosinophils and 47.4% macrophages (**Fig. 2A**) with 2.1% neutrophils and 0.7% lymphocytes (**Fig. 2C**). Treatment with H89 decreased eosinophil numbers by 80%, neutrophil numbers by 64% and lymphocyte numbers by 74% without any effect on macrophage (**Fig. 2A & 2C**). In the moderate model, the cell infiltrate consisted of 39.3% eosinophils, 58.5% macrophages, 1.9% neutrophils and 0.3% lymphocytes and was entirely inhibited by H89 (**Fig. 2B & 2D**).

### Effect of H89 on Peribronchiolar Inflammation and Mucus Production in the Lung

**Figure 3:**
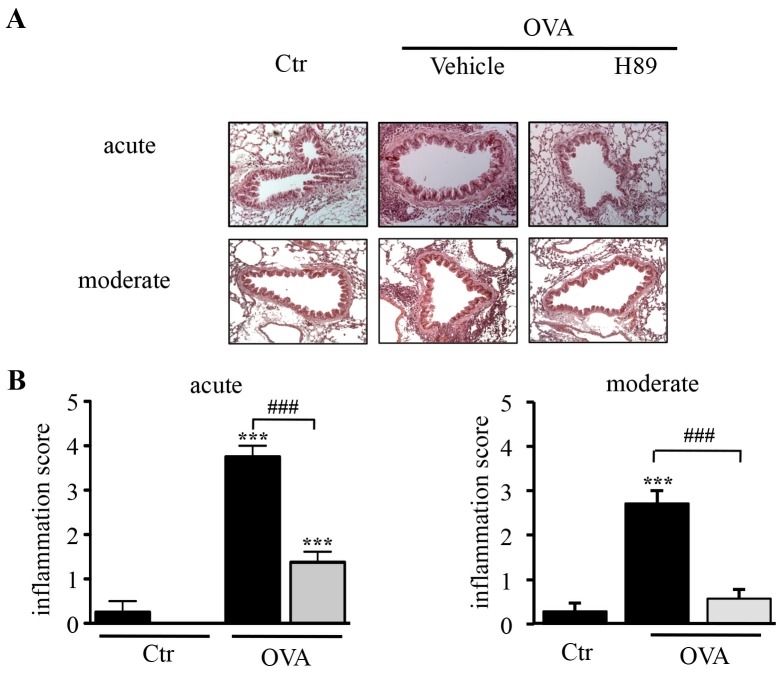
Effect of H89 on lung tissue inflammatory cell infiltration. A. H&E-stained lung sections demonstrating peribronchial inflammatory infiltrates 24 hours after the last OVA challenge (magnification × 200). (**B–C**) Inflammation score in lung sections from control (Ctr) and OVA sensitized/challenged (OVA) mice treated with vehicle (black blocks) or H89 (grey blocks) in the acute (**B**) and moderate (**C**) asthma models. Data represent mean values ± SEM (bars) from *n* = 6 mice per group. ***P<0.001 *vs* corresponding controls; ^###^ *P*<0.001 *vs* group indicated.

Lung tissues collected 24 h after the last challenge showed marked peribronchiolar inflammation in both acute and moderate asthma models (**Fig. 3A**). H89 inhibited this infiltration of inflammatory cells by 63 and 79% in the acute and moderate models, respectively (**Fig. 3B**).

**Figure 4:**
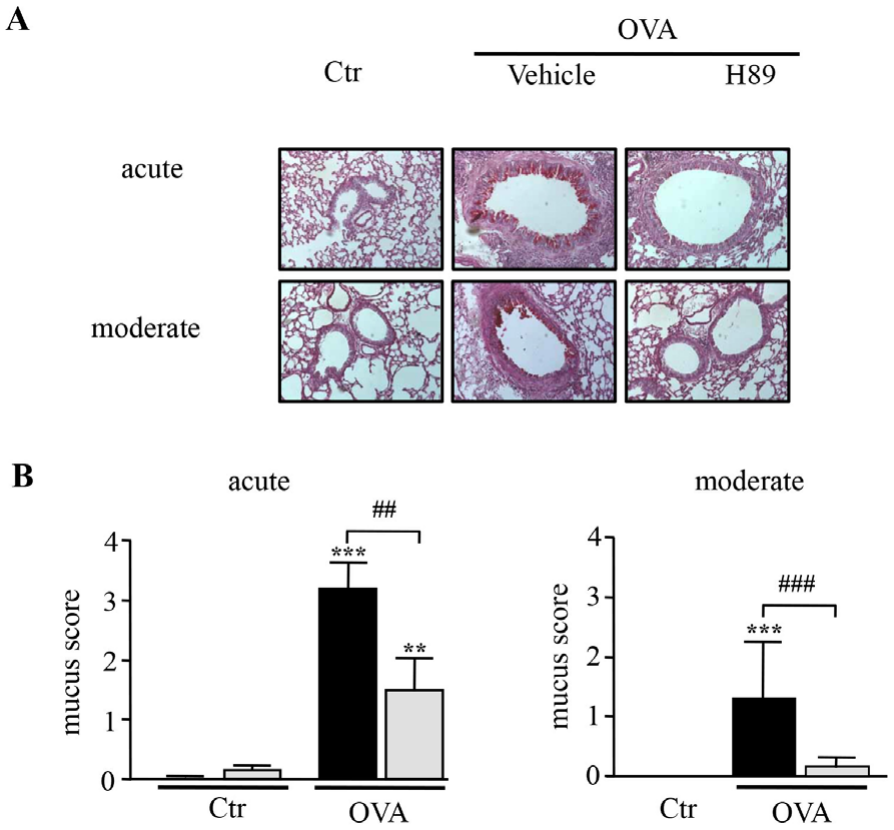
Effect of H89 on mucus cell hyperplasia in the lung. A. Periodic acid Schiff (PAS)-stained lung sections demonstrating hyperplasia of mucus-producing goblet cells 24 hours after the last OVA challenge (magnification × 200). (**B–C**) Mucus score in lung sections from control (Ctr) and OVA sensitized/challenged (OVA) mice treated with vehicle (black blocks) or H89 (grey blocks) in the acute (**B**) and moderate (**C**) asthma models. Data represent mean values ± SEM (bars) from *n* = 6 mice per group. **P<0.01 and ***P<0.001 *vs* corresponding controls; ^##^ *P*<0.01 and ^###^ *P*<0.001 *vs* group indicated.

Periodic acid-Schiff (PAS) staining revealed a strong goblet cell hyperplasia and mucus overexpression in OVA sensitized/challenged mice in both models (**Fig. 4A**). This feature was also significantly inhibited by H89, by 53% and 88% in the acute and the moderate models, respectively (**Fig. 4B**).

### Effect of H89 on Mast Cell Numbers in the Lung

**Figure 5:**
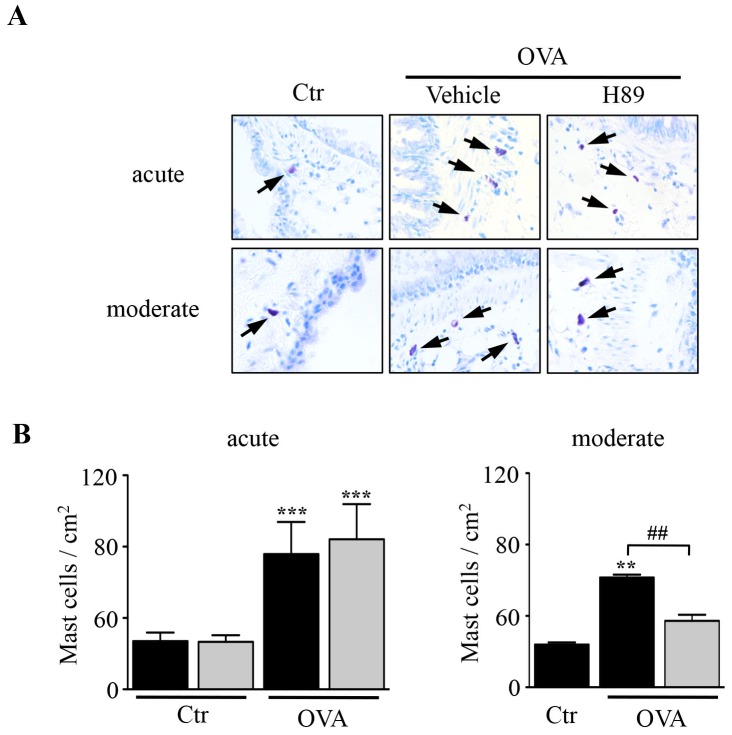
Effect of H89 on mast cell numbers in the lung. A. Acidic toluidine blue-stained lung sections 24 hours after the last OVA challenge (magnification × 200). Black arrows indicate toluidine blue-positive mast cells (**B–C**) Quantification of mast cell numbers in lung sections from control (Ctr) and OVA sensitized/challenged (OVA) mice treated with vehicle (black blocks) or H89 (grey blocks) in the acute (**B**) and moderate (**C**) asthma models. Data represent mean values ± SEM (bars) from *n* = 6 mice per group. **P<0.01 and ***P<0.001 *vs* corresponding controls; ^##^ *P*<0.01 *vs* group indicated.

OVA sensitized/challenged mice displayed a significant increase in lung mast cell numbers in both models as compared to control mice (3.4-fold and 2.6-fold for the acute and the moderate model, respectively) (**Fig. 5A & 5B**). Treatment with the AGC kinase inhibitor H89 significantly reduced lung mast cell numbers in the moderate model, but remained without any effect in the acute model (**Fig. 5A & 5B**).

### Effect of H89 on OVA-induced Th2 Cytokine Production in BAL

**Figure 6:**
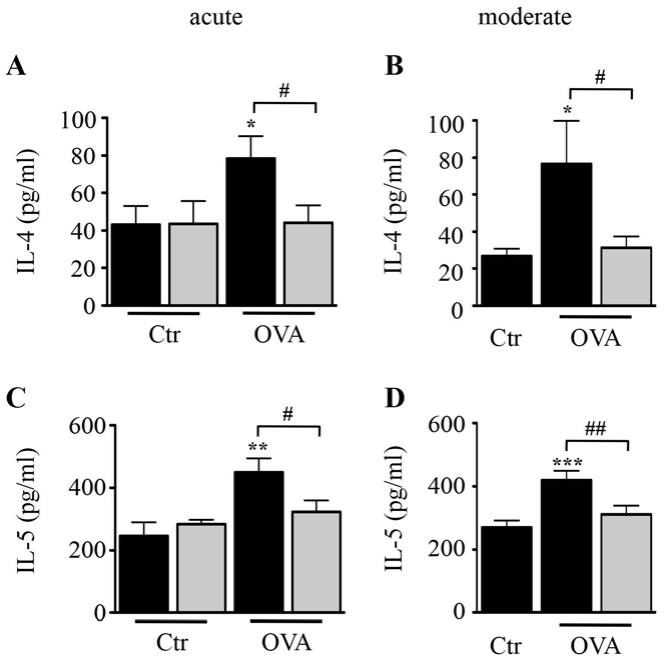
Effect of H89 on Th2 cytokine levels in BAL fluid. BAL fluid was collected 24 hours after the last OVA challenge. The levels of IL-4 (**A–B**) and IL-5 (**C–D**) were determined using ELISA in the acute (**A & C**) and moderate (**B & D**) asthma models in control (Ctr) and OVA-sensitized/challenged (OVA) mice treated with vehicle (black blocks) or H89 (grey blocks). Data represent mean values ± SEM (bars) from *n* = 6−8 mice per group. *P<0.05, **P<0.01 and ***P<0.001 *vs* corresponding controls; ^#^ *P*<0.05 and ^##^ *P*<0.01 *vs* group indicated.

We next assessed the levels of Th2 cytokines IL-4 and IL-5 in BAL fluid collected 24 h after the last challenge. OVA-treated mice showed significantly increased levels of IL-4 and IL-5 in both asthma models. These increases were abolished by H89 in both models (**Fig. 6A, B, C, D**), without modifying baseline levels of control mice (**Fig. 6A & 6C**).

### Effect of H89 on OVA-induced Immunoglobulin (Ig) Levels

**Figure 7:**
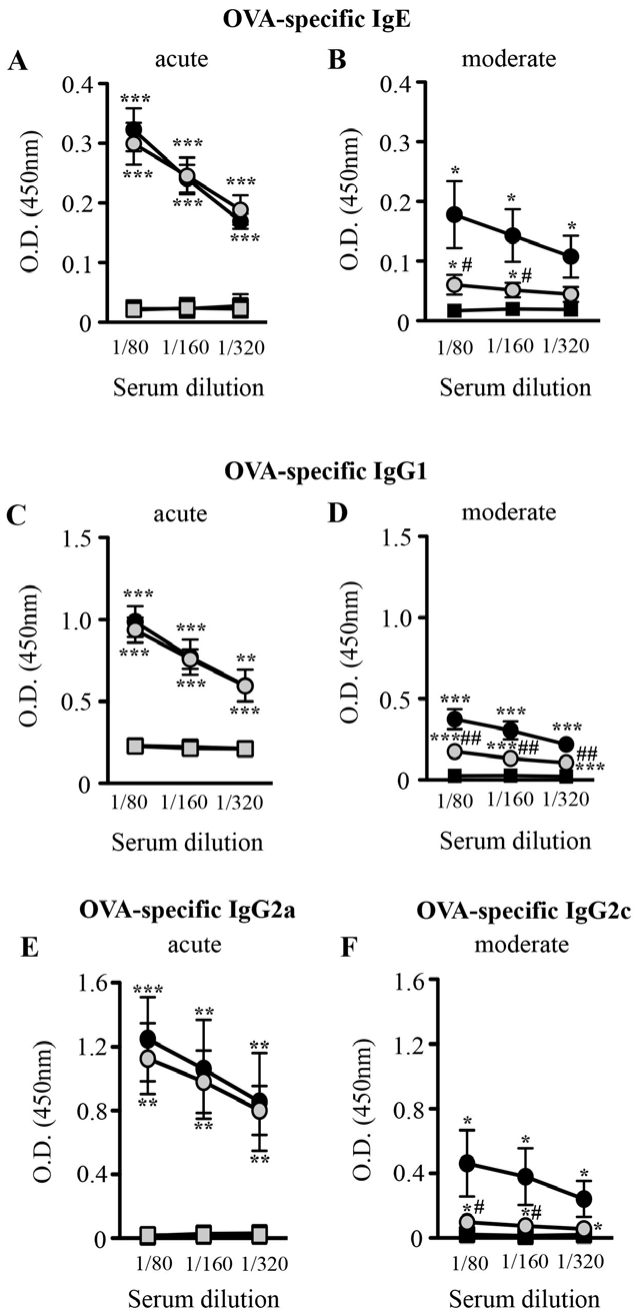
Effect of H89 on serum OVA-specific Ig levels. Serum was collected 24 hours after the last OVA challenge. The levels of OVA-specific IgE (**A–B**), OVA-specific IgG1 (**C–D**), OVA-specific IgG2a (**E**) and OVA-specific IgG2c (**F**) were determined using ELISA in the acute (**A, C & E**) and moderate (**B, D & F**) asthma models in control (squares) and OVA sensitized/challenged (circles) mice treated with vehicle (black blocks) or H89 (grey blocks). Data represent mean values ± SEM (bars) from *n* = 4−6 mice per group (saline) and *n* = 6−12 mice per group (OVA). *P<0.05, **P<0.01 and ***P<0.001 *vs* corresponding controls; ^#^ *P*<0.05 and ^##^ *P*<0.01 *vs* group indicated.

Serum was collected 24 h after the last OVA challenge. OVA treatment led to a significant increase in OVA-specific IgE, IgG1 and IgG2a/c levels, as compared to control mice in both asthma models (**Fig. 7**). Treatment with H89 significantly reduced both OVA-specific IgE, IgG1 and IgG2c levels in the moderate model (**Fig. 7B, 7D & 7F**). By contrast, H89 had no effect on serum OVA-specific Ig-production in the acute model (**Fig. 7A, 7C & 7E**).

We also measured IgA levels in BAL fluids collected 24 h after the last challenge with OVA (**Fig. S1 and Methods S1**). OVA treatment led to a significant increase in total IgA levels, as compared to control mice in both asthma models (**Fig. S1A & S1B**). This increase was totally inhibited by treatment with H89 in both models (**Fig. S1A & S1B**). We also observed a significant increase in OVA-specific IgA in the acute model, but not in the moderate model, and this increase was partially reversed by treatment with H89 (**Fig. S1C & S1D**).

## Discussion

*N*-[2-(*p*-Bromocinnamylamino)ethyl]-5-isoquinolinesulfonamide) (H89) was first described more than 20 years ago as an inhibitor of Protein Kinase A (PKA) [11]. It was later reported to also inhibit Mitogen- and Stress-Activated protein Kinase 1 (MSK1) with a potency similar to that for PKA, and showed some selectivity for other members of the AGC kinase family, including p70 ribosomal protein S6 kinase 1 (S6K1) and Rho-associated kinase (ROCK)-II [14], [15]. H89 has been used extensively *in vitro* as a PKA and MSK1 inhibitor but, to the best of our knowledge, its anti-inflammatory potential *in vivo* has not been reported to date. The present study demonstrates that H89 exerts strong anti-inflammatory properties in mouse models of asthma.

Several models of allergic asthma have been developed in mice. Although these models promote a T helper type 2 (Th2) cell–biased pulmonary inflammation, apparent disparities exist reflecting differences in the strains of mice examined and/or in the protocols used for antigen sensitization and challenge [32], [33]. For these reasons, we decided to assess the anti-inflammatory properties of H89 using two models of allergic asthma and two commonly used strains of mice. The ‘acute’ model we used consisted of sensitization of Balb/c mice by intraperitoneal (i.p.) injection of chicken egg ovalbumin (OVA) adsorbed on the adjuvant aluminum hydroxide (alum), followed by repetitive intranasal (i.n.) challenges with OVA. The use of alum promotes a strong eosinophilic inflammation in the lung, and airway hyperresponsiveness (AHR) that are however independent of IgE production, B cells or mast cells [34]. The ‘moderate’ model we used in C57BL/6 mice consisted of i.p. sensitization with OVA in the absence of adjuvant, followed by i.n. challenges with OVA. Although we did not observe the development of any significant AHR in these conditions, adjuvant-free models of asthma reproduce several features of human asthma including the inflammatory infiltrate and are fully dependent on mast cells when performed in mice on the Th1-prone C57BL/6 background [24], [25] but not in mice on the Th2-prone Balb/c background [35].

We show that H89 is a potent inhibitor of AHR associated with the acute asthma model. Lung inflammation, mucus production and infiltration of eosinophils were reduced by treatment with H89 in both asthma models. Interestingly, treatment with H89 totally inhibited macrophage recruitment in BAL fluid in the moderate model without any effect on infiltrated macrophages in the acute model, suggesting their mode of activation is different. We also observed low but significant numbers of neutrophils and lymphocytes in BAL fluid, whose recruitment was also blocked by H89 in both models. These anti-inflammatory properties of H89 most likely occurred through suppression of Th2 cytokine production as demonstrated here for IL-4 and IL-5 measured in BAL fluid, which is in agreement with findings reporting that H89 can inhibit IL-5 promoter activity and IL-5 production by Th2 cells *in vitro* [22]. Treatment with H89 likely modulates expression of many other inflammatory genes in the lung. For example, Kawaguchi and collaborators showed that treatment of the human bronchial epithelial cell line, BEAS-2B, can suppress IL-17F-induced IL-11 production *in vitro* [36] and we previously reported that H89 can reduce the release of the main mast cell growth factor stem cell factor (SCF) from human lung fibroblasts in primary culture [12].

In addition, we show here an immunomodulatory effect of H89 inhibiting the rise of OVA-specific IgE, IgG1 and IgG2c in the moderate mast cell-dependent model, without any effect in the acute, adjuvant-helped and mast cell-independent condition. By contrast, as concerning IgA production, H89 significantly reduced total IgA levels in BAL fluids in both asthma models, as well as the increased OVA-specific IgA levels in BAL fluids from OVA-treated mice in the acute model. OVA-specific IgA were not enhanced in the moderate model and H89 did no show any effect. Such a limitation of the immune response in these asthma models is a new effect of this AGC kinase inhibitor H89.

The *in vitro* profile of H89 suggests several potential new targets in asthma. Among those, PKA and MSK1 both appear very attractive considering their central role in regulating the activity of pro-inflammatory transcription factors implicated in asthma, in particular NF-κB [12], [13], [16], [17]. Inhibition of the NF-κB pathway reduces inflammation in asthma models [37], [38], [39], [40], and several inhibitors of IKK2/IKKβ, an upstream kinase of NF-κB activation, have been successfully tested preclinically [41]. MSK1 is activated by the p38 and ERK MAP kinases [18] and similarly to NF-κB, several MAP kinase inhibitors are at different stages of preclinical testing for asthma [7].

In addition, MSK1 might even be implicated, at least in part, in the anti-inflammatory properties of glucocorticoids through a mechanism involving a glucocorticoid receptor-dependent export of MSK1 from the nucleus to the cytoplasm [42]. Moreover, H89 potentiates the inhibitory effects of glucocorticoids on TNF-stimulated gene expression *in vitro*, an effect that the authors attributed to inhibition of MSK1 rather than PKA [43].

Surprisingly however, MSK proteins were recently shown to limit proinflammatory signaling ‘downstream’ of Toll-Like Receptors (TLRs) through a mechanism involving induction of expression of the MAP kinase phosphatase (MKP)-1 [44] and IL-1 receptor antagonist (IL-1Ra) [45]. In agreement with these results, MSK1/2 knockout mice showed increased inflammation compared with wild-type mice in a model of oxazolone-induced allergic contact dermatitis [46]. These reports also show that MSK knockout mice have reduced IL-10 expression under inflammatory conditions [44], [45]. However, we did not observe any effect of H89 on IL-10 expression in BAL fluid from OVA sensitized/challenged mice in the two asthma models we used (data not shown).

We previously showed that H89 can directly inhibit NF-κB activation in primary human lung fibroblasts stimulated with IL-1β *in vitro*, through a mechanism involving suppression of MSK1-mediated phosphorylation of the NF-κB subunit p65 at serine 276 [12]. We show here that H89 can also suppress IL-1β-mediated release of the NF-κB -dependent gene IL-6 from peritoneal macrophages *ex vivo* (**Fig. S2A and Methods S1**), suggesting a role for H89 on the inflammatory macrophage phenotype.

OVA-treatment increased the number of mast cells in both asthma models. Interestingly, H89 treatment had no effect on lung mast cell numbers in the acute model where AHR and lung inflammation can develop in the absence of mast cells as shown from studies in mast cell-deficient animals [25]. By contrast, H89 significantly reduced lung mast cell numbers in the moderate asthma model which is highly dependent on the presence and activation of mast cells for airway inflammation and remodeling [24], [25]. We show that treatment of bone marrow-derived cultured mast cells (BMCMCs) with H89 does not inhibit antigen- and IgE-induced mast cell degranulation and IL-6 production *in vitro* (**Fig. S2B & S2C and Methods S1**). Thus it is likely that the decrease in mast cell numbers observed in H89-treated mice in the moderate model reflects the lower levels of IgE in these animals and the subsequent reduced IgE-dependent mast cell activation rather than direct effects of H89 on mast cells.

We finally show that although H89 is a potent anti-inflammatory drug when administered before each challenge, a single treatment with H89 before the last challenge has no effect on AHR or numbers of inflammatory cells in BAL fluids in the acute asthma model (**Figure S3 and Methods S1**). As a positive control, we included a group receiving a single administration of the clinically efficient glucocorticoid dexamethasone (DEX). Although single treatment with DEX was less efficient than treatment with DEX before each challenge, as we reported previously [27], it reduced AHR (although the difference did not reach significance) and slightly but significantly reduced numbers of eosinophils in BAL fluid (**Figure S3**).

In conclusion, we here demonstrate that the AGC kinase inhibitor H89 inhibits airway inflammation and hyperresponsiveness in two murine models of asthma when administered before each challenge. Although particular care must be taken when attempting to extrapolate findings from animal models of a disease to their human counterparts, our results suggest that H89 or other AGC kinase inhibitors might be candidates for alternate treatment in glucocorticoid-resistant asthma patients. One could imagine that a combination of H89 or other AGC kinase inhibitors with glucocorticoids could allow the use of lower drug exposure, and thus reduce adverse events associated to the chronic use of glucocorticoids in asthma.

## Supporting Information

Figure S1 **Effect of H89 on total and OVA-specific IgA levels in BAL fluid.** BAL fluid was collected 24 hours after the last OVA challenge. The levels of total IgA (**A–B**) and OVA-specific IgA (**C–D**) were determined using ELISA in the acute (**A & C**) and moderate (**B & D**) asthma models in control (Ctr) and OVA-sensitized/challenged (OVA) mice treated with vehicle (black blocks) or H89 (grey blocks). Data represent mean values ± SEM (bars) from *n* = 4−8 mice for control groups and *n* = 8−14 mice for OVA sensitized/challenged groups (OVA). *P<0.05, **P<0.01 and ***P<0.001 *vs* corresponding controls; ^#^ *P*<0.05 *vs* group indicated.
(TIF)

Figure S2 **H89 reduces IL-1β-induced macrophage activation but not IgE- and antigen-mediated mast cells activation. A**. Peritoneal macrophages were pre-incubated with H89 (10 mM) or vehicle (DMSO, <0.01%) for 30 min before addition of IL-1β (final concentration: 1 ng/ml) or medium alone for control samples. IL-6 levels were measured by ELISA in the supernatant 6 h after stimulation with IL-1β. **B–C**. Bone marrow-derived cultured mast cells (BMCMCs) were loaded with anti-DNP IgE (1 mg/ml) overnight. Cells were then washed and pre-incubated with H89 (10 µM) or vehicle (DMSO, <0.01%) for 30 min before addition of antigen (DNP-HSA) at the indicated concentration or PMA + A23187 (as a positive control for mast cells degranulation). Mast cell degranulation was assessed by measuring β-hexosaminidase release after 1 h of stimulation with DNP-HSA (**B**), and IL-6 levels were measured by ELISA in the supernatant 6 h after stimulation with DNP-HSA (**C**). All data are means ± SEM from three separate experiments performed in duplicate. *P<0.05, **P<0.01 and ***P<0.001 *vs* corresponding controls; ^#^ *P*<0.05 *vs* group indicated.
(TIF)

Figure S3 **Efficiency of a single administration of dexamethasone or H89 before the last challenge in the acute asthma model.** **A.** Penh responses to aerosolized methacholine in control mice (square) and OVA sensitized-challenged mice (circle) treated with a single i.p. injection of vehicle (black), H89 (10 mg/kg) (grey) or dexamethasone (DEX) (1 mg/kg; Sigma) (white) 2 h before the last OVA challenge in the acute asthma model. **B–C**. Effect of H89 (10 mg/kg) (grey blocks), DEX (1 mg/kg) (white blocks) or vehicle (black blocks) on total leukocyte (Total), eosinophil (Eos) and macrophage (Mac) numbers (**B**), or neutrophils (Neu ) and lymphocytes (Lym) numbers (**C**) in BAL fluid 24 hours after the last challenge in control (Ctr) and OVA-sensitized/challenged mice (OVA) in the acute asthma model. Data represent mean values ± SEM from *n* = 6−12 mice per group (control) and *n* = 12 mice per group (OVA). **P<0.01 and ***P<0.001 *vs* corresponding controls; ^##^ *P*<0.01 and ^###^ *P*<0.001 *vs* group indicated.
(TIF)

Methods S1 Supplementary methods.
(DOCX)
