# Supplementary figures and images for: The AGC Kinase Inhibitor H89 Attenuates Airway Inflammation in Mouse Models of Asthma
Source: PLoS One. 2012 Nov 26;7(11):e49512. doi: 10.1371/journal.pone.0049512 (PMC3506657; doi:10.1371/journal.pone.0049512)

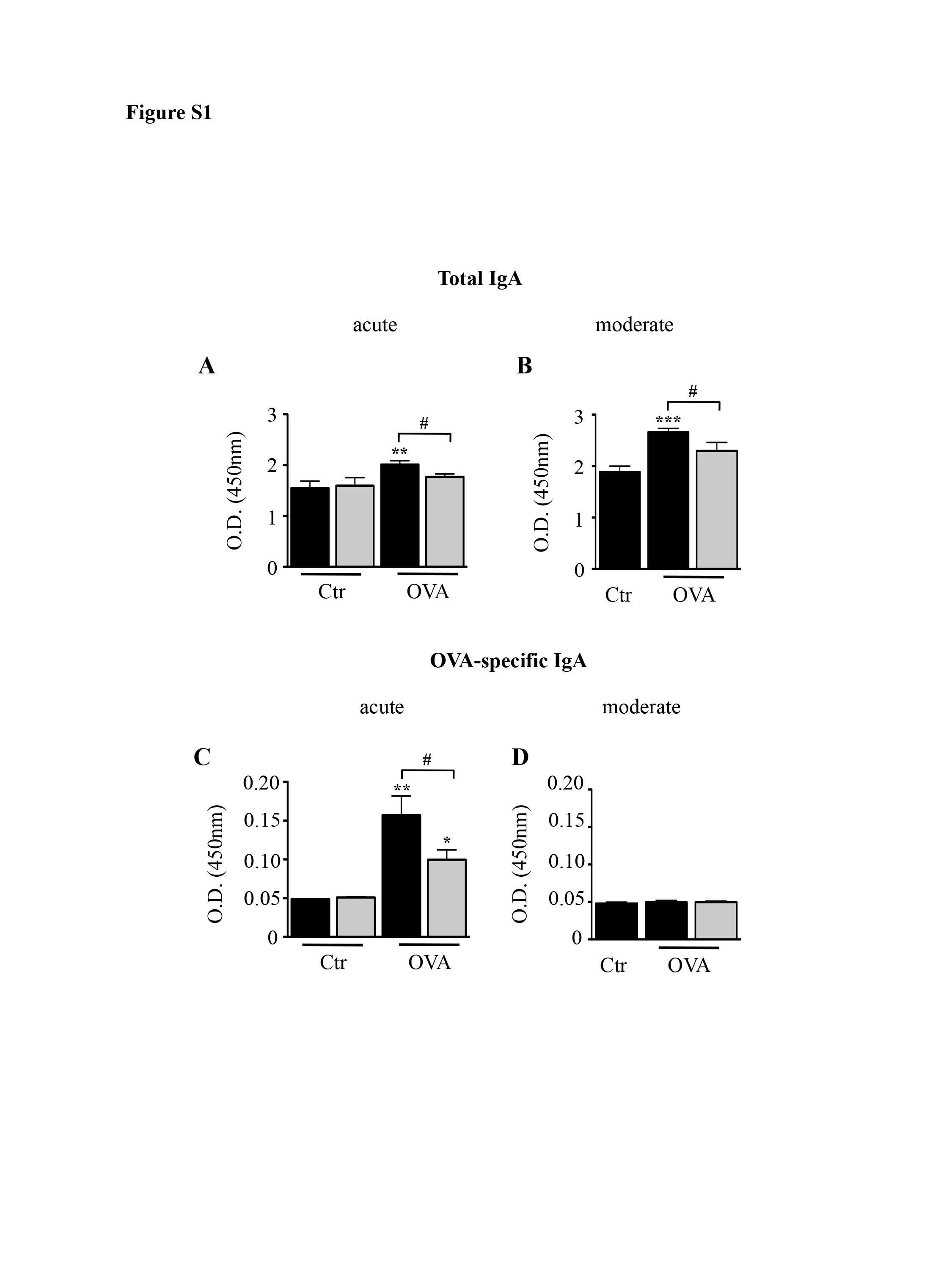

Supplement: Figure S1 — Effect of H89 on total and OVA-specific IgA levels in BAL fluid. BAL fluid was collected 24 hours after the last OVA challenge. The levels of total IgA (A–B) and OVA-specific IgA (C–D) were determined using ELISA in the acute (A & C) and moderate (B & D) asthma models in control (Ctr) and OVA-sensitized/challenged (OVA) mice treated with vehicle (black blocks) or H89 (grey blocks). Data represent mean values ± SEM (bars) from n = 4−8 mice for control groups and n = 8−14 mice for OVA sensitized/challenged groups (OVA). *P<0.05, **P<0.01 and ***P<0.001 vs corresponding controls; # P<0.05 vs group indicated. (TIF) [file pone.0049512.s001.tif]

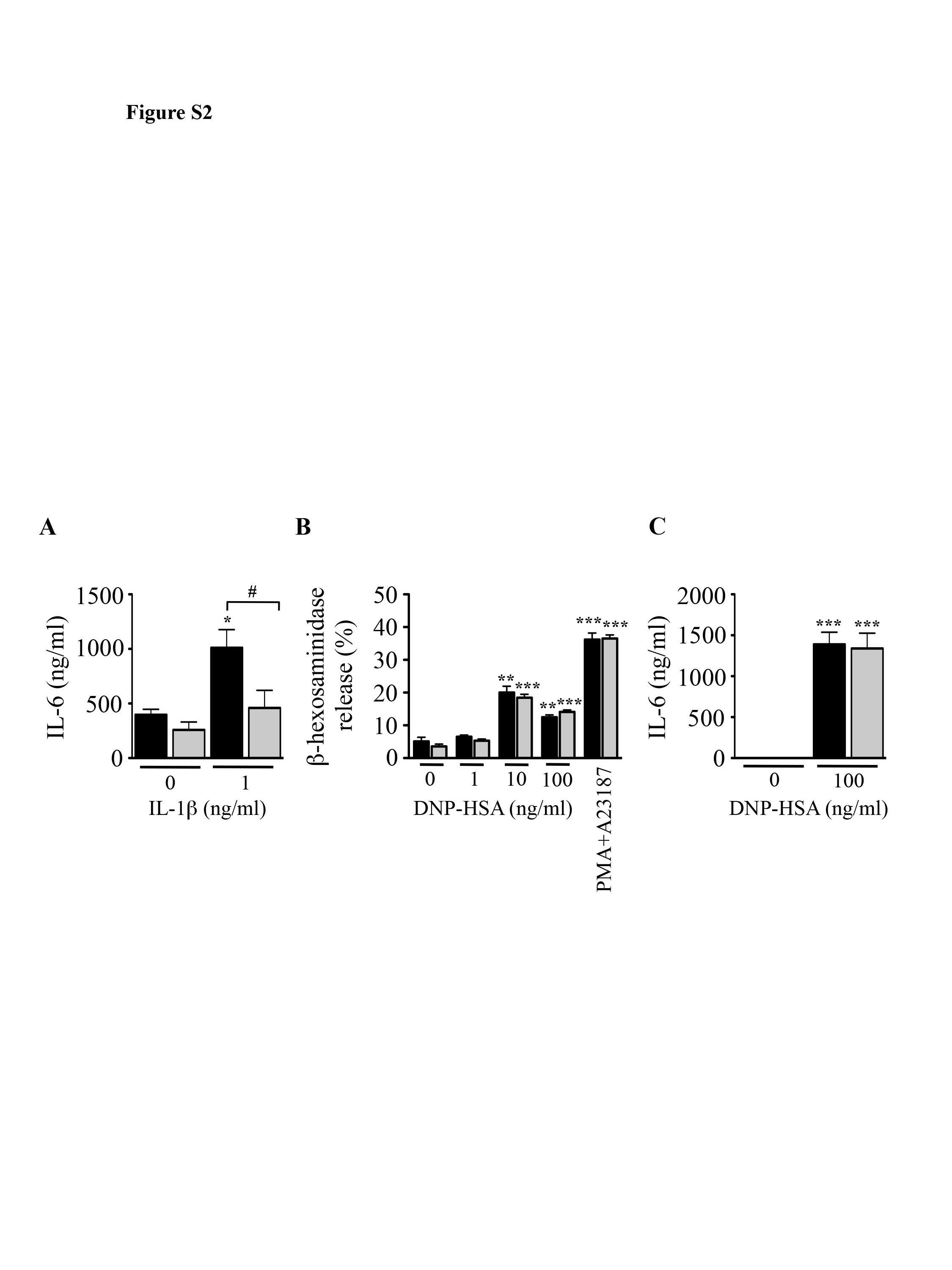

Supplement: Figure S2 — H89 reduces IL-1β-induced macrophage activation but not IgE- and antigen-mediated mast cells activation. A. Peritoneal macrophages were pre-incubated with H89 (10 mM) or vehicle (DMSO, <0.01%) for 30 min before addition of IL-1β (final concentration: 1 ng/ml) or medium alone for control samples. IL-6 levels were measured by ELISA in the supernatant 6 h after stimulation with IL-1β. B–C. Bone marrow-derived cultured mast cells (BMCMCs) were loaded with anti-DNP IgE (1 mg/ml) overnight. Cells were then washed and pre-incubated with H89 (10 µM) or vehicle (DMSO, <0.01%) for 30 min before addition of antigen (DNP-HSA) at the indicated concentration or PMA + A23187 (as a positive control for mast cells degranulation). Mast cell degranulation was assessed by measuring β-hexosaminidase release after 1 h of stimulation with DNP-HSA (B), and IL-6 levels were measured by ELISA in the supernatant 6 h after stimulation with DNP-HSA (C). All data are means ± SEM from three separate experiments performed in duplicate. *P<0.05, **P<0.01 and ***P<0.001 vs corresponding controls; # P<0.05 vs group indicated. (TIF) [file pone.0049512.s002.tif]

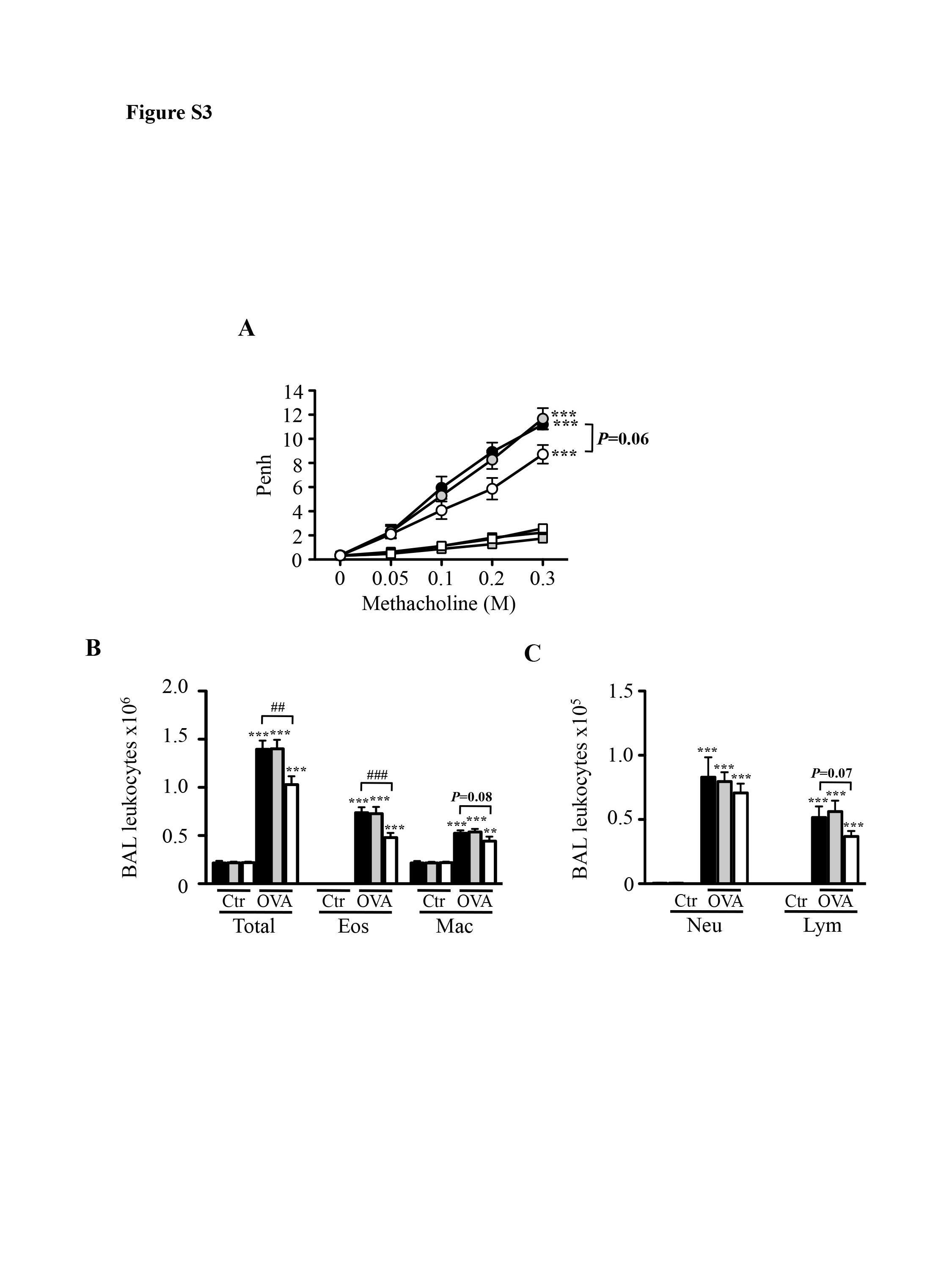

Supplement: Figure S3 — Efficiency of a single administration of dexamethasone or H89 before the last challenge in the acute asthma model. A. Penh responses to aerosolized methacholine in control mice (square) and OVA sensitized-challenged mice (circle) treated with a single i.p. injection of vehicle (black), H89 (10 mg/kg) (grey) or dexamethasone (DEX) (1 mg/kg; Sigma) (white) 2 h before the last OVA challenge in the acute asthma model. B–C. Effect of H89 (10 mg/kg) (grey blocks), DEX (1 mg/kg) (white blocks) or vehicle (black blocks) on total leukocyte (Total), eosinophil (Eos) and macrophage (Mac) numbers (B), or neutrophils (Neu ) and lymphocytes (Lym) numbers (C) in BAL fluid 24 hours after the last challenge in control (Ctr) and OVA-sensitized/challenged mice (OVA) in the acute asthma model. Data represent mean values ± SEM from n = 6−12 mice per group (control) and n = 12 mice per group (OVA). **P<0.01 and ***P<0.001 vs corresponding controls; ## P<0.01 and ### P<0.001 vs group indicated. (TIF) [file pone.0049512.s003.tif]
