# Supplementary material for: The AGC Kinase Inhibitor H89 Attenuates Airway Inflammation in Mouse Models of Asthma
Source: PLoS One. 2012 Nov 26;7(11):e49512. doi: 10.1371/journal.pone.0049512 (PMC3506657; doi:10.1371/journal.pone.0049512)
Supplement: Methods S1 — Supplementary methods. (DOCX) [file pone.0049512.s004.docx]

**Supplementary methods**

***Measurement of total and OVA-specific IgA antibodies***

Total IgA levels were determined by ELISA in bronchoalveolar lavage (BAL) fluid collected 24 hours after the last OVA challenge. Microtiter plates were coated overnight at 4 °C with rat anti-mouse IgA antibodies (C10-3 from BD Biosciences) and blocked for 1 h with 10% bovine serum albumin in phosphate buffered saline. BAL samples were then incubated 2 hours at room temperature. After three washings with PBS containing 0.05% Tween-20, a biotinylated anti-mouse IgA (for measurement of total IgA; C10-1 from BD Biosciences) or a biotinylated anti-OVA antibody (for measurement of OVA-specific IgA) was added to the wells and incubated for 2 h at room temperature. The plates were then washed and incubated with an extravidin-horseradish peroxidase conjugate (Sigma) for 30 min at room temperature. After final washings, the horseradish peroxidase substrate, tetramethylbenzidine (Pharmingen) was added and coloration was allowed to develop for 15–20 min. The reaction was stopped by addition of 0.5 M H_2_SO_4_ and absorbance intensity was read at 450 nm.

***Generation of bone marrow-derived cultured mast cells (BMCMCs)***

Bone marrow cells derived from 10-week-old C57BL/6 mice were cultured in DMEM medium, supplemented with 10% fœtal calf serum (FCS), penicillin and streptomycin and 10 ng/ml recombinant murine IL-3 (R&D). Cells were cultured for 6 weeks, with medium changed twice a week until >95% were FcεRIα^+^KIT^+^ (assessed by Flow cytometry, data not shown).

***Stimulation of BMCMCs with IgE and antigen (DNP)***

BMCMCs were incubated overnight with 2 μg/ml IgE (anti-DNP, clone SPE-7, Sigma) at a concentration of 10^6^ cells/ml at 37°C. Cells were washed to remove unbound IgEs and pre-incubated at 37^o^C with vehicle (DMSO, <0.05%) or H89 (10μM, LC laboratories) in DMEM medium (for assessment of cytokine production) or Tyrode’s buffer (for assessment of mast cell degranulation). After 30 min, BMCMCS were stimulated with various concentrations of DNP or 25 ng/ml PMA + 5μM A23187 (all from Sigma) for 1 h (for assessment of mast cell degranulation) or 6 h (for assessment of cytokine production) at 37^o^C.

***Mast cell degranulation assay (β-hexosaminidase release)***

BMCMCs were stimulated with DNP or PMA as described above. After 1 h of stimulation, cells were centrifuged and the cell pellet lysed with 0.5% Triton-X100 in Tyrode’s buffer. Supernatant and lysates were incubated with 1.3 mg/mL p-nitrophenyl-N-acetyl β-D-glucosamine (Sigma) in 0.1M sodium citrate (pH 4.5) at 37^o^C for 1 h. The reaction was stopped by addition of 0.2M glycine and the enzyme activity was evaluated by measuring optical density at 405 nm. The percentage of specific β-hexosaminidase release was calculated as follows: percentage release = 100 x supernatant activity/ (supernatant activity + cell lysate activity).

***Culture of peritoneal macrophages and stimulation with IL-1β***

To harvest peritoneal cells, 5 ml of PBS buffer were injected into the peritoneal cavity, and the abdomen was gently massaged for 30 sec. Fluid containing peritoneal cells was aspirated, and the cells were centrifuged and resuspended at 10^6^ cells/ml in RPMI medium supplemented with 10% fœtal calf serum (FCS), penicillin and streptomycin and incubated at 37^o^C. Non-adherent cells were removed after 2 hours and adherent macrophages were pre-incubated with vehicle (DMSO, <0.05%) or H89 (10μM, LC laboratories). After 30 min, recombinant murine IL-1β (R&D) was added at a final concentration of 1 ng/ml and the cells were incubated at 37^o^C. After 6 hours, the supernatant was saved at -20^o^C until IL-6 measurement.

***Quantification of IL-6 levels***

IL-6 levels were quantified using an ELISA kit (eBioscience) according to the manufacturer’s instructions.
